# Annotations

**Published:** 1904-10-15

**Authors:** 


### Annotations.

### The Suppression of Noises.

The Society which exists for the restriction of street noises in London must surely command the sympathy of that large army of workers whose living depends on the undisturbed efforts of their brains. Perhaps the triumphs of the Society have not been very considerable, but this may in part be due to the apathy of many for whose benefit it exists. Yet there are unfortunately indications that though the noises of the day, in their most crude form, may be to some extent brought under control, there are others which are decidedly on the increase. It can hardly have escaped attention that, during the last few years, mechanical ingenuity has been directed to the production of instruments capable of emitting musical sounds by an automatic arrangement, or by a method which involves no technical ability on the part of the performer. These instruments may be made to grind out noise by the hour, and their popularity is undoubtedly spreading. Formerly the production of noises of this order was limited by the necessity for the expenditure of time and skill in the training of the performer, but now it appears any one can "play the piano" or other similar instrument by merely working a pedalling arrangement similar to what is used in the case of a sewing machine. In addition there are various "phones" which automatically recite a programme of tunes in a plaintive croak which is said to exercise a peculiar charm on the happiness of the domestic circle. All this is very serious for the brain worker who is so unfortunate as to dwell in the neighbourhood of those who cultivate a passion for the mechanical production of more or less rhythmical sounds. The freedom of the subject may be quoted in defence of those who delight to work a mechanical piano or to listen to hoarse noises emerging from a brazen funnel; but, after all, the neighbourhood has rights as well as the individual. An organ-grinder in the street can be suppressed as a nuisance; why not, then, the similar performer next door? One is prepared to allow full credit to the grateful and refining influence of music, but it occupies a subordinate position relative to the work of the world, and its easy mechanical production on a large scale is calculated to create dismay in the study and the library. If not regulated or suppressed it may well become an intolerable nuisance.

### Some London Statistics.

The report issued by the Public Control Department of the London County Council contains some very impressive figures. Even medical men may be surprised to find that the inquests held during the year ending March 31 numbered no less than 7,245. Among these there were 490 cases of infants suffocated in bed and 334 in which death was attributed to excessive drinking. The number of inquiries into the deaths of children under one year of age was 1,927. Adding the various charges involved in the inquests the cost to the public comes out at nearly £30,000. Another series of figures deals with the action of the Council's inspectors under the Infant Life Protection Act, and it appears that something like 1,000 infants have been under supervision in notified houses. The number of deaths among these children has been 61 out of a total of 972. The Act empowering the Council to make bye-laws for regulating the employment of children generally and for dealing with street trading by persons under 16 years of age has only recently come into operation, so that its value cannot yet be determined, but as it is estimated that the number of school children engaged in paid employment is not less than 50,000 there will be a large opportunity for the practice of an active discretion. The Shop Hours Act, which regulates the employment in shops of persons under 18 years of age, can boast an increasing success, and the number of cases of infringement of its provisions has steadily declined: such is the virtue of an Act of Parliament supported by efficient inspection. The monthly return of the number of paupers in London is a much less satisfactory document. The figures for August show a total of 110,267, which is a ratio of 23·7 per 1,000 inhabitants, and forms the highest percentage for the time of year since August 1871. Reviewing the statistics of pauperism over a long series of years, however, there is no doubt of a very large shrinkage in the proportion of the population in receipt of parochial relief, and the recent rise of the figures must be attributed to some temporary influence of the effect of which, according to the prophets, the approaching winter is to give us still sadder testimony.

### Doctors and Dress.

At the opening of the winter session of the London School of Medicine for Women, Miss Murdoch having dwelt upon the fact that they stepped to-day with ease into some of the finest laboratories in London, and all the recent equipments of science, "an inheritance undreamt of by the women of forty years ago," told them that they would have to face, in the hospital especially, the great realities of life, and that though they would lose many illusions, they would gain many new truths. She proceeded to give the students a number of practical hints, dealing especially with the subject of dress. She advised them to eschew uncommon forms of clothing, and to do their best to dissipate the idea that medical women are careless about their personal attire, or afford excuse for the accusation that they are "frumps." The accusation, she asserted, is false, and we could easily instance well-known medical women whose costume is without reproach. Those who imagine that slovenly dress is associated in the public mind with cleverness are certainly under a delusion; it rather, on the contrary, suggests that the slovenliness extends to other and more important matters. Of course any patient would prefer to be professionally attended by a badly dressed woman whose treatment might be relied upon to a smartly attired woman who did not understand the case. But there is no reason why medical skill and due care of attire should not go together, whether in respect to women or men. Male practitioners are not always free from the reproach of paying inadequate attention to dress; and the slovenly-looking medical man may be seen both in cities and in country villages. It is not the first of their duties to wear immaculate frock coats, but for the credit of their profession, as well as for their own sake, they should dress in a manner that does not invite, if it does not justify, invidious criticism.

